# Bibliometric analysis of the inflammation expression after spinal cord injury: current research status and emerging frontiers

**DOI:** 10.1038/s41393-024-01038-w

**Published:** 2024-10-03

**Authors:** Xiaoyu Li, Kun Jiao, Chen Liu, Xiongfei Li, Shanhe Wang, Ye Tao, Yajun Cheng, Xiaoyi Zhou, Xianzhao Wei, Ming Li

**Affiliations:** Department of Orthopedics, Shanghai Changhai Hospital, Shanghai, China

**Keywords:** Neuroimmunology, Regeneration and repair in the nervous system

## Abstract

**Study design:**

Bibliometric analysis.

**Objective:**

To analyze literature on inflammatory expression following spinal cord injury, highlighting development trends, current research status, and potential emerging frontiers.

**Setting:**

Not applicable.

**Methods:**

Articles were retrieved using terms related to spinal cord injury and inflammatory responses from the Web of Science Core Collection, covering January 1, 1980, to May 23, 2024. Tools like CiteSpace and VOSviewer assessed the research landscape, evaluating core authors, journals, and contributing countries. Keyword co-occurrence analyses identified research trends.

**Results:**

A total of 2504 articles were retrieved, showing a consistent increase in publications. The Journal of Neurotrauma had the highest publication volume and influence. The most prolific author was Cuzzocrea S, with Popovich PG having the highest H-index. China led in the number of publications, followed closely by the United States, which had the highest impact and extensive international collaboration. Research mainly focused on nerve function recovery, glial scar formation, and oxidative stress. Future research is expected to investigate cellular autophagy, vesicular transport, and related signaling pathways.

**Conclusion:**

The growing interest in inflammation caused by spinal cord injury is evident, with current research focusing on oxidative stress, glial scar, and neurological recovery. Future directions include exploring autophagy and extracellular vesicles for new therapies. Interdisciplinary research and extensive clinical trials are essential for validating new treatments. Biomarker discovery is crucial for diagnosis and monitoring, while understanding autophagy and signaling pathways is vital for drug development. Global cooperation is needed to accelerate the application of scientific findings, improving spinal cord injury treatment.

## Introduction

Spinal cord injury (SCI) is a central nervous system disorder characterized by high morbidity and mortality rates [1, 2] resulting in the loss of sensory, motor, and autonomic functions below the injury level. Moreover, SCI presents numerous severe complications, such as pressure ulcers [3] and urinary tract infections [4, 5]. According to global statistics, the incidence of SCIs is approximately 105 cases per million [6], with the United States and China having approximately 54 [7] and 49.8 [8] reported cases per million, respectively. Notably, these figures display an increasing trend every year [9].

Spinal cord injuries can be categorized as primary and secondary injuries based on their pathogenic processes [10]. Primary injuries result from mechanical trauma, such as traffic accidents, falls, sports activities, or acts of violence [11], and are irreversible. In contrast, secondary injuries arise from the foundation of primary injuries, with pathological processes, including vascular damage [12], ischemic edema [13], oxidative stress [14], apoptosis [15], glial scarring [16], and Wallerian degeneration [17]. These processes are reversible. The inflammatory response plays a crucial role in secondary SCI, is mediated by microglia [18], lymphocytes [19], and neutrophils [10], and may compromise the blood-spinal cord barrier [20, 21]. Concurrently, microglia induce astrocyte differentiation toward the A1 subtype [22], amplifying the inflammatory response and directly or indirectly causing the death of neuronal cells, ultimately affecting motor function recovery.

To date, many studies have explored the inflammatory response following SCI, providing a foundation for treating this condition. However, for researchers to obtain a comprehensive understanding of the value of specific literature is difficult; as the significant contributions made by countries, institutions, and authors; the current research hotspots within the field; and future trends. Bibliometrics enables the analysis of the development and research patterns of scientific investigations over a specific period and facilitates the prediction of potential future research directions. Therefore, in this study, we aimed to utilize bibliometric methods to investigate the current status and frontiers of research on inflammation after SCI.

## Methods

### Data sources

After obtaining relevant keywords and supplementing them with MeSH subject headings sourced from PubMed, a search was performed through the Web of Science Core Collection database using the following search strategy: (TS = ((“Spinal Cord*“ OR “Myelopath*“) AND (“Injur*“ OR “ Trauma*“ OR “Contusion*“ OR “Laceration*“ OR “Transection*“ OR “Post Traumatic”))) AND TS = (“Inflammatory Response*“). The search conducted on May 30, 2024, at 16:24:32 (GMT + 0800, CST), encompassed articles from January 1,1980, to May 23, 2024. In total, 2504 articles related to the expression of inflammation after SCI were observed.

### Methodology

The raw data were initially imported into Microsoft Excel 2019 (Microsoft Corp., Redmond, WA, USA) for initial organization and collation. Subsequently, the Bibliometrix package in R 4.3.0 (R Software for Statistical Computing, Vienna, Austria) was employed for publication statistics and analysis of journal provenance. National collaboration analyses were conducted using VOSviewer 1.6.19 (Leiden, The Netherlands) and Scimago Graphica. CiteSpace 6.3.R1 was used for author and institutional collaboration analyses as well as co-occurrence, clustering, and bursting analyses of keywords. Given that the H-index reflects a researcher’s impact by indicating they have published at least H papers, each of which has been cited at least H times in other publications [23], we used it to measure the scientific influence of scholars.

## Results

### Trends of publications

**Fig. 1:**
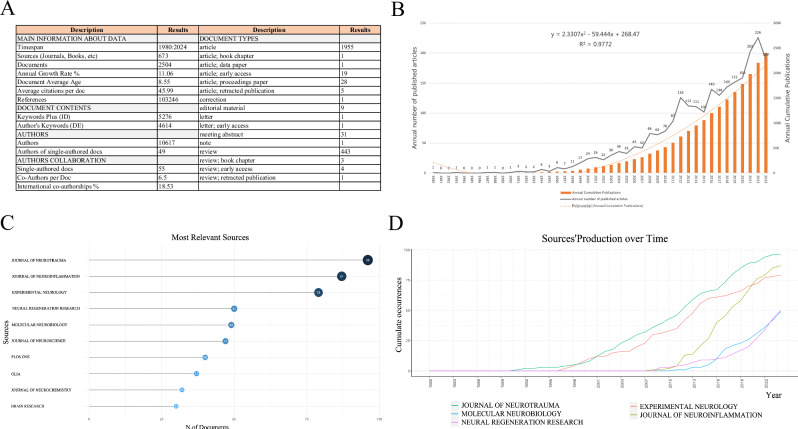
The publication details, volumes, and leading journals are summarized as follows. **A** Summary of general details of publications; **B** Annual publication volume and annual cumulative publication volume; **C** Top 10 journals by publication volume; **D** Top 5 journals bycumulative publication growth.

A total of 2504 studies focusing on inflammation after SCI were published. As displayed in Fig. 1B, a noticeable upward trend in the cumulative and annual publication of articles was observed, indicating a growing scholarly interest in this research topic. The earliest relevant article was published in 1980 and a substantial increase was observed in annual publications from 1995 to 2012. Although certain years witnessed a slight decline (2013–2015), the number of publications presented an upward trend after 2016, reaching a peak in 2022, with 226 publications.

### Analysis of high co-occurrence journals

As displayed in Fig. 1A, literature regarding the expression of inflammation after SCI has been disseminated across 673 journals. As illustrated in Fig. 1C, the top 10 journals collectively published 547 papers, representing approximately 21.85% of the total publications. Notably, the *Journal of Neurotrauma* published the highest number of papers (96 articles). Figure 1D displays the top 5 journals exhibiting the most rapid growth in cumulative publications over time, with the *Journal of Neurotrauma* leading the pack. This suggests that the journal is experiencing substantial growth and recognition in its respective fields.

### Analysis of author collaboration

**Fig. 2:**
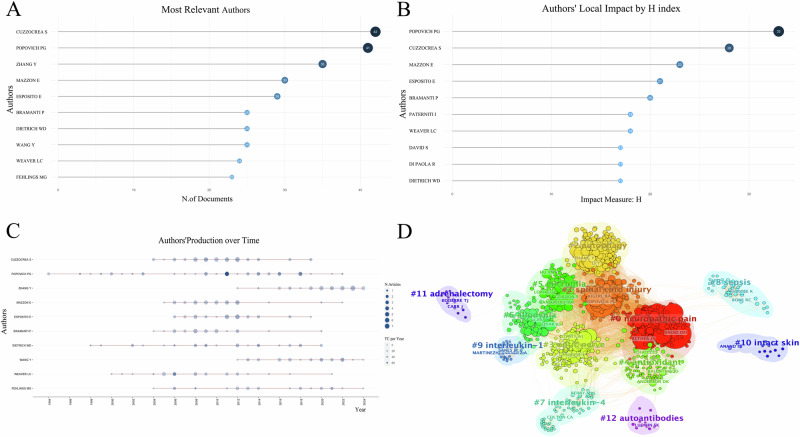
The top authors by publication volume, H-index impact, production over time, and cluster analysis are as follows. **A** Top 10 authors by publication volume; **B** Top 10 authors by H-index with the highest local impact; **C** Top 10 authors’ production over time; **D** Author cluster analysis.

A total of 10,617 authors contributed to the writing and publishing of the relevant literature. As displayed in Fig. 2A, the top three authors in terms of publications were Cuzzocrea S (42 articles), Popovich PG (41 articles), and Zhang Y (35 articles). As shown in Fig. 2B, we listed the top 10 authors in H-index, among which Popovich PG ranked first with H = 33, Cuzzocrea S ranked second with H = 28, Mazzon E ranked third with H = 23. As depicted in Fig. 2C, the size of each node represents the annual output of individual authors, while the depth of color indicates the annual total citations for each author. Over time, there is a noticeable increase in citations related to inflammation expression after SCI, suggesting a growing interest among scholars in this field. Network diagrams of author cluster analysis (Fig. 2D) were generated using CiteSpace, revealing the formation of numerous academic clusters in this research area. Cluster analysis reveals that academic clusters centered around scholars like Anderson DK and Chao C focus on oxidative stress following spinal cord injury (SCI). For example, Anderson DK explores the potent antioxidant and cytoprotective effects of manganese superoxide dismutase (MnSOD) in SCI [24]. Chao C reviews how hydrogen gas exerts antioxidative, anti-inflammatory, and anti-apoptotic effects by selectively scavenging harmful oxygen radicals in SCI [25]. In contrast, clusters led by scholars like Ransohoff and Loane DJ focus more on microglial changes post-SCI. Loane DJ discusses the activation of microglia after SCI, highlighting their role in releasing pro-inflammatory and anti-inflammatory cytokines and regulating glial scar formation [26]. Ransohoff explores how inhibiting CX3CR1 expression can reduce the recruitment and activation of pro-inflammatory microglia, thereby enhancing recovery after SCI [27]. Another academic cluster, centered around scholars like Zhang Y and Li Y, mainly focuses on autophagy following SCI. Li Y examines the role of autophagy in SCI and its impact on microglia-mediated neuroinflammation and motor function [28]. Zhang Y studies how Resveratrol activates the NRF2/HO-1 signaling pathway [29], and how Modified Black Phosphorus Quantum Dots downregulate the mTOR signaling pathway [30], both of which promote autophagy, leading to the clearance of metabolic waste and damaged organelles in injured cells.

### Analysis of institutional cooperation

**Fig. 3:**
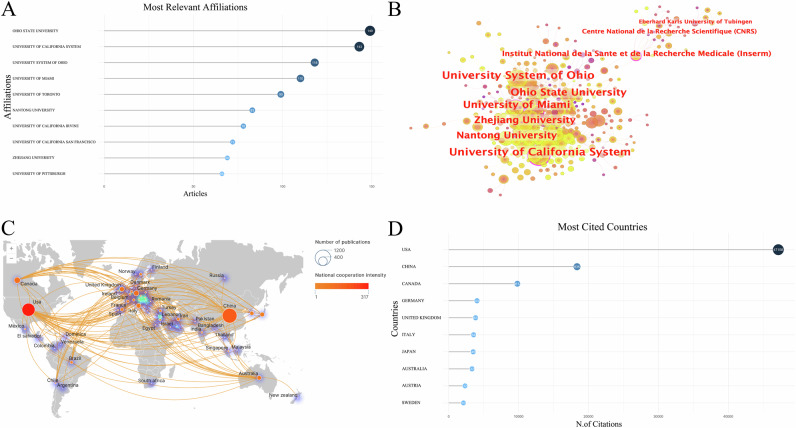
The top affiliations and countries by publication volume and number of citations are as follows. **A** Top 10 affiliations by publication volume; **B** Institution cooperation analysis; **C** National cooperation analysis; **D** Top 10 most cited countries.

A total of 2035 institutions contributed to the publication of research articles in this field. As shown in Fig. 3A, Ohio State University (149 articles), the University of California System (143 articles) and the University System of Ohio (118 articles) published the largest number of articles. These three institutions have demonstrated significant collaborations with other institutions, with the University of California System standing out as a key influencer in this field (Fig. 3B).

### Analysis of country cooperation

Fifty-five countries have published studies on the expression of inflammation after SCI. China (974 articles), the United States (765 articles), and Canada (173 articles) were ranked among the top three countries in terms of publications (Fig. 3C). The United States exhibited the highest level of collaboration with other countries and was the most frequently cited country (Fig. 3D). China had the highest publication volume, however, the intensity of China’s collaboration with other countries is relatively weak. Additionally, the density surrounding the circle indicates that Europe currently serves as the center of research in this field.

### Keyword analysis

**Fig. 4:**
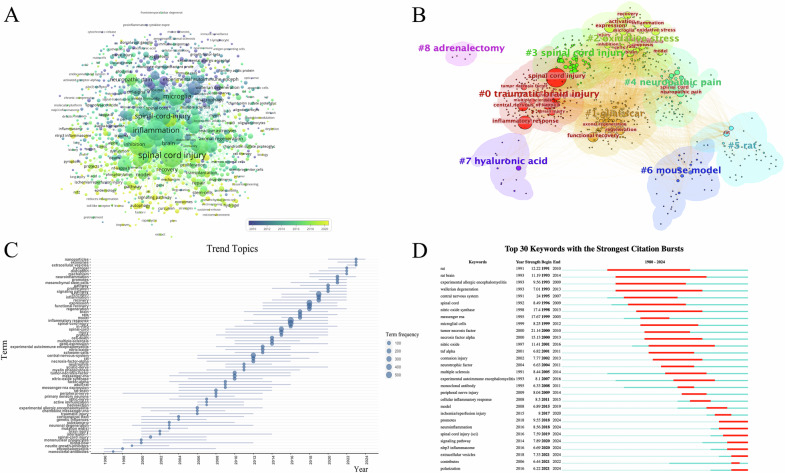
The analysis of keywords by temporal distribution, clustering, trend topics, and citation bursts is as follows. **A** Temporal distribution analysis of keywords; **B** Clustering analysis of keywords; **C** Analysis of trend topics; **D** Strongest citation bursts analysis of keywords.

A total of 5276 keywords were identified, with the top five being inflammatory response, spinal cord injury, expression, activation, and functional recovery (Fig. 4A). The aforementioned keywords were categorized into nine clusters, primarily focusing on six aspects (Fig. 4B): traumatic spinal cord or brain injury, oxidative stress, glial scars, neuropathic pain, rat or mouse model, and others. Figure 4C displays that topics, such as extracellular vesicle, hydrogel and neuroinflammation, have gained significant attention recently and are emerging as research hotspots in this field. Furthermore, we utilized CiteSpace to extract citation bursts for all keywords, with a specific focus on the top 30 keywords. NLRP3 inflammasome is an important component of the immune system, playing a crucial role in inflammatory response and cellular autophagy. Keywords related to NLRP3 and extracellular vesicles continue to appear until 2024 (Fig. 4D), indicating that these areas are ongoing research priorities and will remain focal points in the field in the coming years.

**Fig. 5:**
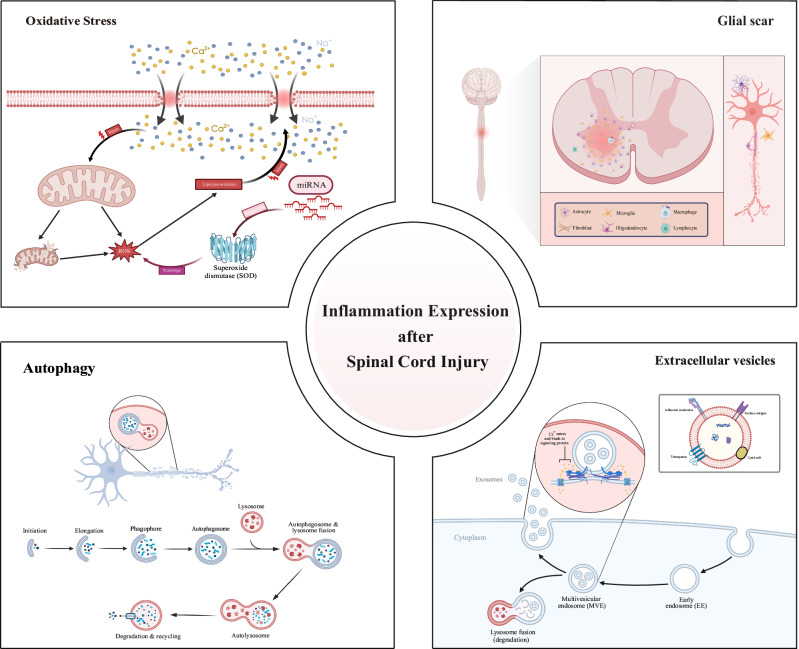
Hotspots and focal point of the research.

Figure 5 provides a comprehensive overview of the hotspots and focal point of the research, which encompasses keywords with 4 clusters, including “oxidative stress”, “glial scars”, “autophagy” and “extracellular vesicles”. SCI can compromise the integrity of cell membranes, allowing extracellular calcium ions to flood into the cell. This calcium overload disrupts mitochondrial function, impairing energy production and reducing the cell’s ability to clear reactive oxygen species (ROS). Meanwhile, mitochondria themselves can trigger increased ROS production through calcium ion stimulation, exacerbating oxidative stress [31]. The excess ROS leads to lipid peroxidation, further damaging cell membranes and allowing even more calcium ions to enter the cell, creating a positive feedback loop that results in cellular dysfunction or death. To counteract this, cells activate mechanisms to eliminate ROS, including the upregulation of superoxide dismutase (SOD) enzyme activity, which converts superoxide anions into less reactive forms. Additionally, microRNAs (miRNAs) may play a regulatory role in this defense process by modulating the expression of SOD and other antioxidant enzymes [32, 33]. After SCI, astrocytes proliferate and migrate to the injury site, where they express intermediate filament proteins such as GFAP, forming a physical barrier. Concurrently, fibroblasts are activated and migrate to the injured area, secreting extracellular matrix molecules like CSPGs and collagen, further reinforcing the scar structure. Microglia and macrophages, as key immune response cells, play a critical role in the formation and maintenance of the glial scar by phagocytosing cellular debris and modulating the inflammatory response [34]. Oligodendrocyte precursor cells migrate to the injury site and differentiate into mature oligodendrocytes, contributing to myelin regeneration [35]. Additionally, monocytes, T cells, and B cells are recruited to the injury site after the trauma, where they release cytokines and chemokines, participating in the formation and maintenance of the glial scar. The interactions among these cells and the exchange of molecular signals collectively drive the formation of the glial scar. After SCI, the critical steps of cellular autophagy include autophagosome formation, fusion, and degradation, all of which are essential for maintaining cellular homeostasis. The autophagy process begins with the formation of an isolation membrane, or phagophore, in the cytoplasm. This membrane then extends and closes with the assistance of various autophagy-related proteins [36], forming a double-membraned structure known as the autophagosome. The autophagosome encapsulates damaged organelles or protein aggregates, and subsequently fuses with a lysosome to create an autolysosome. Within the autolysosome, acidic hydrolases break down the encapsulated contents, thereby enabling the recycling of cellular materials [37]. The biosynthesis of exosomes involves the encapsulation of various biomolecules, including proteins, lipids, and nucleic acids [38]. Exosomes are formed within the cytoplasm and develop from early endosomes within multivesicular bodies. They can carry signaling proteins and tetraspanins, and participate in intercellular interactions through lipid rafts. Following SCI, exosomes can transport molecules like miRNA, which directly bind to target mRNA, promoting its degradation and suppressing the expression of target genes. This regulation influences processes such as inflammation, apoptosis, microglial polarization, and autophagy [39].

## Discussion

### Analysis of research trends

This study utilized bibliometrics to analyze articles published over the past 40 years on post-SCI inflammatory expression. The findings revealed that the number of published articles varied, with an average of less than 10 per year before 1997, but exceeded 200 in the past 3 years, the average annual growth rate is 11.06%. This indicates that the field has garnered significant attention from researchers and has established a solid foundation for future investigations.

Among the prominent journals in this field, the *Journal of Neurotrauma, Journal of Neuroinflammation*, and *Experimental Neurology* stand out with a high number of articles. Notably, the *Journal of Neurotrauma* had the greatest number of articles and demonstrated the fastest growth rate in article publications. Therefore, researchers are suggested to submit relevant papers to these journals as they may serve as core publications in the field.

### Collaborative network analysis

In this field, a wide range of contacts and collaborations among different authors, institutions, and countries exist. Regarding the authors of the articles, the majority (94.32%) had published only three or fewer articles, suggesting that most researchers had not yet conducted extensive studies. Additionally, the field has immense potential and warrants further exploration by researchers. Among the authors, Prof. Salvatore Cuzzocrea from the Institute of Pharmacology, University of Messina, Italy, has the highest number of publications (42 articles). The H-index indicates that researchers have published at least H papers, each of which has been cited at least H times in other publications [23, 40]. Therefore, we use it to reflect the research influence of scholars. Among them, Popovich PG from the Department of Neuroscience, Ohio State University, USA, ranked first with H = 33. Authors such as CUZZOCREA S and POPOVICH PG have consistently maintained high productivity over an extended period, with their research widely cited, demonstrating significant influence in their respective fields. Conversely, authors like MAZZON E and ESPOSITO E have published numerous high-quality articles within a shorter timeframe, highlighting their remarkable contributions during critical phases of current research in their field. The researchers in this field are recommended to pay attention to articles authored by the aforementioned individuals. In addition, cluster analysis revealed the formation of multiple thematic clusters, with neuropathic pain [41], spinal cord injury [42], autophagy [43], and microglia [44] emerging as the most concentrated areas of research. The intersections and correlations among various research topics highlight the interdisciplinary and collaborative nature of modern scientific inquiry. Based on these findings, it is recommended to strengthen cooperation among authors from different fields and to promote interdisciplinary research within this domain. Establishing interdisciplinary research teams and integrating professional knowledge and technical methods from various disciplines can address complex scientific challenges in the study of inflammatory expression following SCI. Additionally, creating an online collaboration platform can facilitate communication and resource sharing among researchers from different fields, enhancing resource sharing and utilization, improving the efficiency of scientific research, and promoting more opportunities for collaboration.

Among the institutions that have made significant contributions in the field of post-SCI inflammation expression, Ohio State University has the highest publication volume and substantial academic influence. The University System of Ohio, University of Miami, Zhejiang University, Nantong University, University of California System, and other institutions are at the center of the cooperation network. These institutions not only publish a large number of research results but also play crucial roles in international collaboration. In terms of specific country contributions, the number of articles published by China and the United States is significantly higher than that of other countries. However, the United States is far ahead in terms of paper citations, and its level of cooperation with other countries is notably higher. Currently, the research center in this field is located in Europe. Given this situation, researchers from different countries should strengthen cooperation to promote the development of this field. By enhancing collaboration, researchers can jointly advance the understanding and progress of inflammation expression after SCI.

In conclusion, the inflammation expression after SCI has garnered significant attention from numerous countries, institutions, and authors. However, the current level of cooperation remains insufficient. Therefore, it is recommended that researchers from various countries further strengthen their collaborative efforts, particularly those institutions at the core of international cooperation networks. By engaging in joint research, the advantageous resources and technologies of various countries can be leveraged to promote the in-depth development of research in the field of post-SCI inflammation expression.

### Current research focus

High-frequency keywords serve as indicators of research hotspots in specific fields of study. High-frequency keywords associated with inflammatory responses after SCI included oxidative stress, glial scars, extracellular vesicle, inflammasome and cellular autophagy. This suggests that the current research emphasizes understanding the above themes. These topics have garnered significant attention and are likely to continue to shape the direction of research in the field.

Oxidative stress is a significant pathophysiological event following injury, playing a crucial role in SCI [45], particularly during the secondary damage phase [46]. Mitochondrial dysfunction is considered a primary source of reactive oxygen species (ROS) in SCI [47, 48]. Post-injury, mitochondria produce excess ROS. Concurrently, factors such as N-methyl-D-aspartate receptor activation [49] and ionic imbalance [50, 51] lead to the accumulation of ROS and reactive nitrogen species [52, 53]. The imbalance between oxidation and antioxidation results in an excess of free radicals. The reaction between ROS and nitric oxide produces large amounts of peroxynitrite, which induces lipid peroxidation, irreversibly damaging neuronal membrane lipids and proteins [54, 55], and triggering neuronal apoptosis and other forms of damage [54].

In order to reduce this damage, researchers have proposed mitochondrial-targeted strategies, such as using mitochondrial protectants to decrease ROS production and enhance mitochondrial function [56]. Antioxidant therapy has shown significant potential in mitigating oxidative stress in SCI. In recent years, various antioxidants have been developed. For example, the traditional drug methylprednisolone [57], has demonstrated antioxidant efficacy in treating SCI but its high doses may lead to severe side effects. Meanwhile, the study of microRNAs (miRNAs) [58, 59] as ROS scavengers has become a research hotspot. By silencing or overexpressing miRNAs the downstream levels of injury-related proteins [60, 61], can be regulated, providing potential therapeutic methods for treating SCI and targeting oxidative stress. However, further research is needed to assess potential adverse effects such as the lack of clinical trial data remains a major limitation. Some novel antioxidants are also being developed and tested, including single-atom cobalt nanozymes [62] and natural antioxidants like melatonin [63]. Single-atom cobalt nanozymes effectively reduce ROS levels and protect spinal cord tissue by mimicking natural enzyme antioxidant functions [62]. Melatonin, as a natural antioxidant, has been extensively studied for its neuroprotective effects in SCI. Melatonin not only reduces oxidative stress but also inhibits inflammatory responses and cell apoptosis [64], thus improving functional recovery after SCI. Melatonin exerts its effects through its receptors, regulating multiple signaling pathways [65] and demonstrating multi-target therapeutic potential. Research on oxidative stress in SCI is ongoing, and antioxidant therapy shows broad application prospects. Future studies will aim to translate these laboratory findings into clinical treatment methods to improve the prognosis for SCI patients.

Glial scars are formed by the proliferation, migration, and differentiation of reactive astrocytes, microglia, and oligodendrocyte precursor cells at the injury site. Glial scars provide structural support and isolate the damaged area to prevent inflammation spread, reducing further damage [16, 66]. However, components in glial scars, such as chondroitin sulfate proteoglycans, release inhibitory signals that hinder the growth and regeneration of nerve axons [67]. After SCI, reactive astrocytes [68] quickly activate and migrate to the injury site, releasing cytokines, chemokines, and growth factors to regulate inflammation and repair processes. Microglia [69], the immune cells of the central nervous system, also rapidly activate after SCI, contributing to inflammation and scar formation. Oligodendrocyte precursor cells differentiate into oligodendrocytes post-injury [70], aiding in myelin regeneration.

In recent years, researchers have explored methods to promote nerve regeneration by inhibiting glial scar formation. For example, inhibiting the JAK/STAT signaling pathway [71, 72] reduces the proliferation of reactive astrocytes and the formation of glial scars, thereby promoting axonal regeneration. Another research direction involves altering the cellular and molecular composition of glial scars to reduce their inhibitory effect on axonal regeneration. For instance, inhibiting the synthesis of chondroitin sulfate proteoglycans [73] can significantly enhance axonal regeneration and functional recovery post-SCI. Additionally, using neurotrophic factors such as BDNF and NT-3 [74] can improve the microenvironment of glial scars, supporting nerve regeneration. Stem cell therapy has also made progress in SCI treatment. Studies have shown that transplanting neural stem cells or induced pluripotent stem cells [75] can generate new neurons and glial cells at the injury site, replacing damaged cells and promoting regeneration. Moreover, these stem cells can secrete various growth factors and anti-inflammatory agents, improving the injury microenvironment and facilitating functional recovery. The application of nanotechnology in SCI treatment is gaining attention [76]. By delivering anti-inflammatory drugs, antioxidants [77], and growth factors through nanoparticles, precise regulation of glial scar formation can be achieved. Future research needs to further optimize these treatment strategies, determining the optimal therapeutic window, dosage, and delivery methods. Large-scale clinical trials are essential to validate the safety and efficacy of these new treatment approaches. By deeply investigating the mechanisms of glial scar formation and developing innovative therapeutic strategies, we aim to provide more effective treatments for SCI patients, ultimately improving their quality of life.

### Future research directions

The exploration of post-SCI inflammation has burgeoned into a vibrant domain within neuroscience, with autophagy and extracellular vesicles emerging as prominent areas of inquiry. A review of frequently cited keywords indicates a sustained academic interest in these subjects for the foreseeable future. In recent years, the number of academic papers related to autophagy and extracellular vesicles has significantly increased, which may be attributed to the attention and recognition of these research fields by the Nobel Prize in Physiology or Medicine. In 2016, the accolade was bestowed upon the Japanese scientist Yoshinori Ohsumi [78] for his groundbreaking contributions to the understanding of cellular autophagy. His work shed light on the lysosome-dependent process of intracellular degradation that is distinctive to eukaryotic cells [79]. Autophagy, a cellular housekeeping mechanism, involves the formation of autophagosomes—double-membrane vesicles that sequester cytoplasmic materials and merge with lysosomes to form autolysosomes, facilitating the degradation of their contents [80]. To date, three types of autophagy have been identified: macroautophagy, microautophagy, and chaperone-mediated autophagy [81]. The plasma membrane’s dynamic elongation and expansion encase various cytoplasmic components within autophagosomes. Autophagy is pivotal for the degradation and recycling of cellular constituents, safeguarding against cellular damage and preserving cellular equilibrium. Moreover, it is responsive to a spectrum of cellular stressors, including hypoxia, starvation, and hyperthermia [82]. Extracellular vesicles, as vital mediators for intercellular communication, are instrumental in the inflammatory response post-SCI. The 2013 Nobel Prize honored the discoveries of vesicle transport regulation mechanisms by James E. Rothman [83], Randy W. Schekman [84], and Thomas C. Südhof [85]. Their research elucidated the fundamental processes and regulatory mechanisms governing vesicle budding, tethering, and fusion, enhancing our comprehension of intracellular trafficking and signal transduction. Investigating signaling pathways is essential for deciphering the transmission and modulation of molecular signals within cells. Disruptions in these pathways are intricately connected to the etiology and progression of numerous diseases. Consequently, the study of these pathways not only unveils fundamental biological principles but may also lay the groundwork for novel preventative and therapeutic approaches. In the context of SCI-induced inflammation, the interplay among autophagy, signaling pathways, and extracellular vesicles presents a compelling avenue for further investigation [86]. Autophagy’s potential to modulate inflammatory responses by influencing signaling pathway components and activities [87], and extracellular vesicles’ capacity to transport inflammatory signaling molecules [88], are mechanisms that could significantly impact the reactions of both distant and neighboring cells. A profounder grasp of these interactions may uncover novel therapeutic targets and pave the way for innovative strategies in SCI management.

Future research may focus on the regulatory mechanisms of autophagy, particularly its role in post SCI inflammation, as well as the complex role of extracellular vesicles in SCI inflammation. The interactions between signaling pathways and their interactions with autophagy and EVs are crucial for understanding inflammation control and nerve healing. Identifying new biomarkers can enhance the early diagnosis, treatment monitoring, and prognosis evaluation of SCI, guiding personalized treatment strategies. Future drug development will depend on a deep understanding of autophagy, EVs, and signaling pathways to create therapies that can regulate inflammation and promote neural recovery. Multi-center clinical trials will ensure the safety and effectiveness of new treatment methods. Meanwhile, interdisciplinary collaboration will utilize diverse expertise to address the challenges of SCI treatment. Global cooperation and knowledge sharing will accelerate the dissemination and application of scientific breakthroughs, promoting SCI treatment research. This collaborative effort may make significant progress in SCI treatment, in order to better improve the prognosis and rehabilitation prospects of patients.

### Strengths and limitations

This study employed bibliometric methods to quantitatively analyze comprehensive information regarding inflammation following SCI. This approach enables researchers to gain a rapid understanding of the current research landscape and identify frontiers in the field. Moreover, the approach lays the foundation for future in-depth research and collaboration between researchers and institutions.

However, the study had some limitations. First, the analysis only considered papers from the Web of Science Core Collection database, potentially overlooking relevant studies from other databases, such as PubMed, Scopus, and Embase. Second, data collection was limited to papers published until May 30, 2024, because the Web of Science Core Collection databases are consistently updated. Therefore, papers published after this date were not included in the analysis.

## Conclusion

The scientific community’s engagement with the inflammatory response post-SCI is witnessing a sustained surge, underscoring an urgent need to bolster collaborative efforts among researchers, institutions, and nations. The prevailing research is concentrated on the regulation of oxidative stress, modulation of glial scar formation, and the restoration of neurological function. Forthcoming research endeavors are likely to concentrate on the intricate roles of autophagy and extracellular vesicles, potentially uncovering groundbreaking therapeutic methodologies. The indispensable role of interdisciplinary research is paramount, as is the imperative to conduct extensive, multicenter clinical trials to substantiate the efficacy of innovative treatment approaches. The discovery of novel inflammatory biomarkers is of vital importance for the prompt diagnosis and persistent monitoring of SCI treatment. Moreover, an enhanced comprehension of the interplay between autophagy, extracellular vesicles, and their signaling pathways is the cornerstone for propelling forward drug development initiatives. Strongly call for strengthening global cooperation and disseminating knowledge to accelerate the practical application of scientific discoveries, thereby accelerating the progress of SCI treatment, with the ultimate goal of improving patient prognosis and enhancing rehabilitation prospects.
